# Assessment of the oncological outcomes of three different bacillus Calmette–Guérin strains in patients with high-grade T1 non-muscle-invasive bladder cancer

**DOI:** 10.1080/2090598X.2021.1874628

**Published:** 2021-01-13

**Authors:** Łukasz Nowak, Wojciech Krajewski, Marco Moschini, Joanna Chorbińska, Sławomir Poletajew, Andrzej Tukiendorf, Tim Muilwijk, Steven Joniau, Alessandro Tafuri, Alessandro Antonelli, Rossella Orlando, Ettore Di Trapani, Mario Alvarez-Maestro, Giuseppe Simone, Stefania Zamboni, Claudio Simeone, Maria Cristina Marconi, Riccardo Mastroianni, Radosław Piszczek, Evanguelos Xylinas, Romuald Zdrojowy, on behalf of European Association of Urology - Young Academic Urologists (EAU-YAU): Urothelial Carcinoma Working Group

**Affiliations:** a Department of Urology and Oncologic Urology, Wroclaw Medical Univeristy, Wroclaw, Poland; b Klinik Für Urologie, Luzerner Kantonsspital, Lucerne, Switzerland; c Second Department of Urology, Centre of Postgraduate Medical Education, Warsaw, Poland; d Department of Public Health, Wrocław Medical University, Wrocław, Poland; e Department of Urology, University Hospitals Leuven, Leuven, Belgium; f Department of Urology, University of Verona, Azienda Ospedaliera Universitaria Integrata Verona, Verona, Italy; g Department of Urology, IEO European Institute of Oncology, IRCCS, Milan, Italy; h Department of Urology , Hospital Universitário La Paz, Madrid, Spain; i Oncologic Urology, ‘Regina Elena’ National Cancer Institute, Department of Urology, Rome, Italy; j Urology Unit, ASST Spedali Civili and Department of Medical and Surgical Specialties, Radiological Science and Public Health, University of Brescia, Brescia, Italy; k Department of Urology and Oncologic Urology, Lowersilesian Specialistic Hospital, Wroclaw, Poland; l Department of Urology, Bichat-Claude Bernard Hospital, Assistance Publique-Hôpitaux De Paris, Paris Descartes University, Paris, France

**Keywords:** Non-muscle-invasive bladder cancer, bacillus Calmette–Guérin, strain, recurrence, progression

## Abstract

**Objective:**

: To determine whether there are significant differences in oncological outcomes between three different bacillus Calmette–Guérin (BCG) strains used for adjuvant intravesical immunotherapy in patients with high-grade T1 (T1HG) non-muscle-invasive bladder cancer (NMIBC).

**Patients and methods:**

: Data of 590 patients with a diagnosis of primary T1HG NMIBC were retrospectively reviewed. The study included 138 (23.4%) patients who were treated with the Moreau, 272 (46.1%) with the TICE, and 180 (30.5%) with the RIVM strains. All patients included in the analysis received at least five instillations of an induction course and at least two installations of a maintenance course. Due to existing differences in baseline patient characteristics, the association between oncological outcomes and strain groups was investigated by complementary analysis with the implementation of inverse probability weighting (IPW).

**Results:**

: The 5-year recurrence-free survival (RFS) rate was 70.5%, 66.7% and 55.2% for the Moreau, TICE and RIVM groups, respectively (*P* = 0.016). The 5-year progression-free survival (PFS) rates were 84.4%, 85% and 77.8% in the Moreau, TICE and RIVM groups, respectively (*P* = 0.215). The IPW-adjusted Cox proportional hazard regression analysis did not show any differences in RFS between the Moreau and TICE groups (*P* = 0.69), whereas the RIVM strain was significantly associated with worse RFS compared to the Moreau (hazard ratio [HR] 1.69 for RIVM; *P* = 0.034) and TICE (HR 1.87 for RIVM; *P* = 0.002) strains. The IPW-adjusted analysis did not show any significant differences between study groups in terms of PFS.

**Conclusions:**

: The results of the present study suggest that the Moreau and TICE strains might be superior to the RIVM strain in terms of RFS in patients with T1HG NMIBC.

**Abbreviations:** CIS: carcinoma *in situ*; IPW: inverse probability weighting; IQR: interquartile range; HR: hazard ratio; HG: high grade; LVI: lymphovascular invasion; MP: muscularis priopria; NMIBC: non-muscle-invasive bladder cancer; PFS: progression-free survival; RCT: randomised controlled trial; RFS: recurrence-free survival; T1HG, high-grade T1; (re-)TURB: (re-staging) transurethral resection of bladder; VH: variant histology

## Introduction

In the management of high-risk (HR) non-muscle-invasive bladder cancer (NMIBC), transurethral resection of bladder (TURB) followed by adjuvant intravesical BCG immunotherapy has been considered the most effective form of treatment [1]. Although BCG has been used as an immunotherapeutic agent for almost 40 years, it is still unknown why treatment results vary among patients [2]. One of the unanswered question remains whether attainable BCG strains are equal in terms of oncological outcomes. International guidelines do not include any clear recommendations regarding the preferable use of certain BCG strains and there are only several direct head-to-head comparisons between commonly used strains in the available literature [1,3,4].

Identification of the most efficient BCG strain and assessing its optimal administration regimen could improve oncological outcomes in patients with NMIBC. Further, due to current global shortages of BCG supplies, possible schedule modification, including the administration of selected strains, has been considered a valuable strategy for treatment optimisation [5].

The aim of the present study was to determine whether there were significant differences in oncological outcomes between three different BCG strains used for adjuvant intravesical immunotherapy in patients with high-grade T1 (T1HG) NMIBC.

## Patients and methods

This retrospective observational cohort study was approved by an Institutional Review Board for institutional data sharing from all of the participating sites. Data of 590 patients with a diagnosis of primary T1HG NMIBCs, treated with maintenance BCG immunotherapy at seven tertiary high-volume care centres between 2001 and 2019 were retrospectively reviewed. Three BCG strains were used: Moreau, TICE, and RIVM. The selection of the strain type resulted primarily from differences in supply and distribution at each participating centre. Therefore, it was not a planned randomised allocation.

The main analysis included 138 (23.4%) patients who were treated with the Moreau strain, 272 (46.1%) with the TICE strain, and 180 (30.5%) with the RIVM strain. Patients included in the analysis received a minimum of five instillations of an induction course and at least two installations of a maintenance course. BCG instillations were given in accordance to international guidelines and local protocols at that time with ≥1 year of planned maintenance. All patients underwent re-staging TURB (re-TURB) performed after initial TURB and before BCG introduction.

The basic sociodemographic characteristics of patients included age at the time of surgery, gender, and smoking history. The available pathological data comprised the tumour stage, tumour grade, tumour size, tumour focality, the presence of concomitant carcinoma *in situ* (CIS), and the presence of muscularis propria (MP) involvement in primary TURB specimen. Data about immediate single-instillation chemotherapy, lymphovascular invasion (LVI), variant histology (VH), and prostatic involvement of the tumours were not uniformly reported, and therefore not included in the analysis.

Surgical specimens were evaluated by dedicated uropathologists in each participating centre without the application of any central assessment protocol. Tumours included in the analysis were staged according to the American Joint Committee on Cancer TNM staging classification and graded according to the WHO 2004 grading system.

There was no standardised follow-up schedule due to the retrospective nature of the study and multicentric data acquisition. Patients were followed-up according to international guidelines at that time.

Concomitant CIS was defined as the coexistence of CIS in conjunction with the exophytic tumour. A recurrence was defined as a recurrence of a tumour of any stage and grade confirmed by TURB and histological or cytological evaluation. Viable tumours at re-TURB and tumour recurrence in the upper urinary tract were not considered as recurrence. Progression was defined as tumour relapse at tumour stage ≥T2 in the bladder or stromal invasion of the prostatic urethra or as distant (e.g. lymph nodes) progression. The patients with T2 lesions at re-TURB were not included in the analysis as they were not suitable for BCG therapy.

The primary database comprised 1511 patients with high-risk NMIBC. The following exclusion criteria were incorporated: incomplete data on major variables; tumours other than T1HG NMIBC; recurrent tumours; incomplete primary TURB and evident residual disease; less than five BCG induction instillations; less than two BCG maintenance installations; a follow-up period of <6 months; other than a full dose concentration of BCG for a given strain; BCG strain other than Moreau, TICE or RIVM; and the modification of the BCG strain during treatment. Finally, 590 cases were included in the analysis.

The primary endpoints of this study were 5-year recurrence-free survival (RFS) and 5-year progression-free survival (PFS).

### Statistical analysis

Descriptive statistics of the categorical variables were presented as counts and percentages. Medians and interquartile ranges (IQRs) were reported for continuous variables. Study groups were compared using chi-squared and Mann–Whitney *U*-tests. Kaplan–Meier curves were plotted for RFS and PFS, and differences between times of survival in each group were evaluated with a likelihood-ratio test. Additionally, Cox regression analyses were performed for both RFS and PFS. Patients without an event or death before an event were censored at the last date of follow-up. Times to events were calculated taking the date of BCG initiation as time zero.

Due to differences in baseline patient characteristics, the association between survival outcomes and particular strain groups was investigated by complementary analysis with the implementation of inversed probability weighting (IPW) adjusted for gender, smoking status, age, the presence of MP in primary TURB specimen, tumour focality, tumour size, and incidence of concomitant CIS [6]. We used IPW-adjusted Cox proportional hazard regression analysis to calculate the IPW-adjusted hazard ratio (HR) and 95% CI for all included strain pairs.

All *P* values were two-sided, with *P* < 0.05 considered statistically significant. Statistical analyses were performed using STATISTICA 13.3 (TIBCO Software Inc., Palo Alto, CA, USA) and the R platform (R project, Vienna, Austria).

## Results

Baseline patient characteristics are presented in table 1. Groups did not differ statistically in terms of gender and the presence of concomitant CIS; however, significant differences in smoking history, tumour size, tumour focality, and the presence of MP in the TURB specimen were observed.

**Table 1.** The patients’ baseline characteristics (chi-squared and Mann–Whitney *U*-test *P* values of the differences between the two study groups)

Variable	All patients *N* = 590	Moreau *n* = 138 (23.4%)	TICE *n* = 272 (46.1%)	RIVM *n* = 180 (30.5%)	*P*
Age, years, median (IQR)	66.9 (58–75)	61.1 (55–66)	71.1 (65–78)	66.1 (56–74)	<0.001a
Gender, *n* (%) Male Female	493 (83.6) 97 (16.4)	114 (82.6) 24 (17.4)	232(85.3) 40 (14.7)	147 (81.7) 33 (18.3)	0.979
Smoking history, *n* (%) Never Former Current Unknown	171 (29) 241 (40.8) 161 (27.3) 17 (2.9)	52 (37.6) 43 (31.2) 43 (31.2) 0 (0)			<0.001a
			63 (23.1) 136 (50) 57 (21) 16 (5.9)	56 (31.1) 62 (34.4) 61 (33.9) 1 (0.6)	
Concomitant CIS, *n* (%) Yes No Unknown	119 (20.2) 466 (79) 5 (0.8)	28 (20.3) 110 (79.7) 0 (0)	46 (16.9) 222 (81.6) 4 (1.5)	45 (25) 134 (74.4) 1 (0.6)	0.654
Tumour size, *n* (%) <3 cm >3 cm Unknown	294 (49.8) 251 (42.6) 45 (7.6)	79 (57.2) 59 (42.3) 0 (0)	123 (45.2) 117 (43) 32 (11.8)	92 (51.1) 75 (41.7) 13 (7.2)	0.021a
Tumour focality, *n* (%) Solitary Multiple Unknown	275 (46.6) 281 (47.6) 34 (5.8)	88 (63.8) 50 (36.2) 0 (0)	100 (36.8) 150 (55.1) 22 (8.1)	87 (48.3) 81 (45) 12 (6.7)	<0.001a
MP in the primary TURB specimen, *n* (%) Yes No Unknown	432 (73.2) 121 (20.5) 37 (6.3)	104 (75.4) 32 (23.2) 2 (1.4)	208 (76.5) 53 (19.5) 11 (4)	120 (66.7) 36 (20) 24 (13.3)	0.005a
Total number of BCG instillations; median (IQR)	15 (9–18)	15 (12–21)	14 (9–18)	15 (11–18)	0.083

**a** Statistically significant *P* value.

The median (IQR) follow-up for the whole population was 40 (25–60) months. The patients were enrolled prospectively and the groups were not matched in terms of observation time. For this reason, our primary analyses were performed for 5-year RFS and PFS.

Recurrence occurred in 37 (26.8%) patients in the Moreau group, 95 (34.9%) in the TICE group, and 71 (39.4%) in the RIVM group (*P* = 0.471). Progression of the cancer was observed in 17 (12.3%) patients in the Moreau group, 39 (14.3%) in the TICE group, and in 30 (16.7%) in the RIVM group (*P* = 0.976). There were 12 (35.3%), 14 (8.7%) and eight (4.4%) cancer-specific deaths in the Moreau, TICE and RIVM groups, respectively (*P* = 0.269)

**Figure 1.**
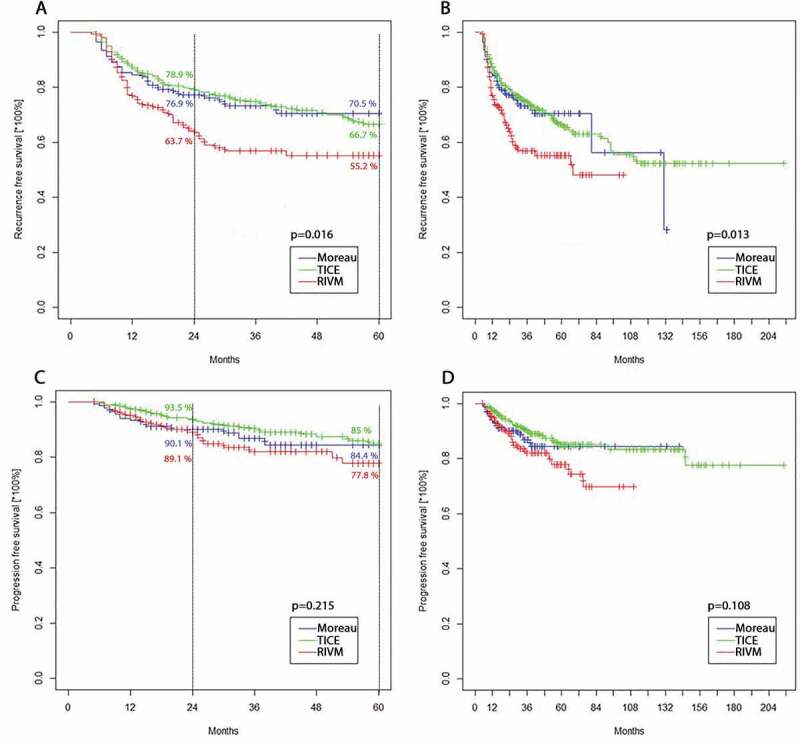
**A**. RFS for the 5-year follow-up (*P* = 0.016). **B**. RFS for the whole observation period (*P* = 0.013). **C**. PFS for the 5-year follow-up (*P* = 0.215). **D**. PFS for the whole observation period (*P* = 0.108)

The 5-year RFS rate was 70.5%, 66.7% and 55.2% for the Moreau, TICE and RIVM groups, respectively. The observed differences in RFS between the study groups were statistically significant for both 5-year (*P* = 0.016) (figure 1A) and the whole observation period (*P* = 0.013) (figure 1B). The 5-year PFS rate was 84%, 85% and 77.8% in the Moreau, TICE and RIVM groups, respectively (figure 1C), being statistically non-significant (*P* = 0.108). No differences were also found for the whole observation period for PFS (*P* = 0.215) (figure 1D).

Considering the IPW-unadjusted multivariate analysis (table 2), the RIVM strain was associated with significantly worse RFS compared to either the Moreau (HR 1.61, 95% CI 1.05–2.47for RIVM; *P* = 0.029) or TICE strains (HR 1.66, 95% CI 1.15–2.38 for RIVM; *P* = 0.006). No other significant differences in both RFS and PFS were found between particular strain groups.

**Table 2.** Multivariable analysis assessing factors associated with RFS and PFS

Variable	Recurrence-free survival (RFS)			Progression-free survival (PFS)		
	*HR*	*95% CI*	*p-value*	*HR*	*95% CI*	*p-value*
Age	0.99	0.98–1.01	0.668	0.99	0.97–1.02	0.579
Gender (**female** vs male)	1.20	0.79–1.81	0.377	1.13	0.58–2.21	0.709
Smoking (never/**any**)	1.13	0.81–1.57	0.475	1.03	0.61–1.73	0.916
MP in primary TURB (**yes**/no)	0.91	0.61–1.32	0.586	0.83	0.47–1.45	0.497
Concomitant CIS (**yes** vs no)	1.58	0.94–2.29	0.081	1.73	0.87–3.55	0.072
Size (≤3 cm vs **>3 cm**)	1.02	0.75–1.39	0.941	1.25	0.76–2.07	0.375
Focality (solitary vs **multiple**)	1.11	0.81–1.51	0.516	1.25	0.75–2.09	0.384
BCG strain: HR (95% CI); p-value	*vs. Moreau*	*vs. TICE*	*vs. RIVM*	*vs. Moreau*	*vs. TICE*	*vs. RIVM*
**Moreau**	X	1.03 (0.68–1.58); p = 0.881	0.62 (0.40–0.95); p = 0.029a	X	1.34 (0.72–2.56); p = 0.357	0.81 (0.42–1.56); p = 0.523
**TICE**	0.97 (0.63–1.47); p = 0.881	X	0.60 (0.42–0.87); p < 0.001a	0.74 (0.39–1.39); p = 0.357	X	0.60 (0.34–1.06); p = 0.081
**RIVM**	1.61 (1.05–2.47); p = 0.029a	1.66 (1.15–2.38); p < 0.001a	X	1.23 (0.64–2.35); p = 0.523	1.65 (0.94–2.91); p = 0.081	X

**a** Statistically significant *P* value. HR >1 worse outcome for the bolded option, HR <1 better outcome for the bolded option.

**Figure 2.**
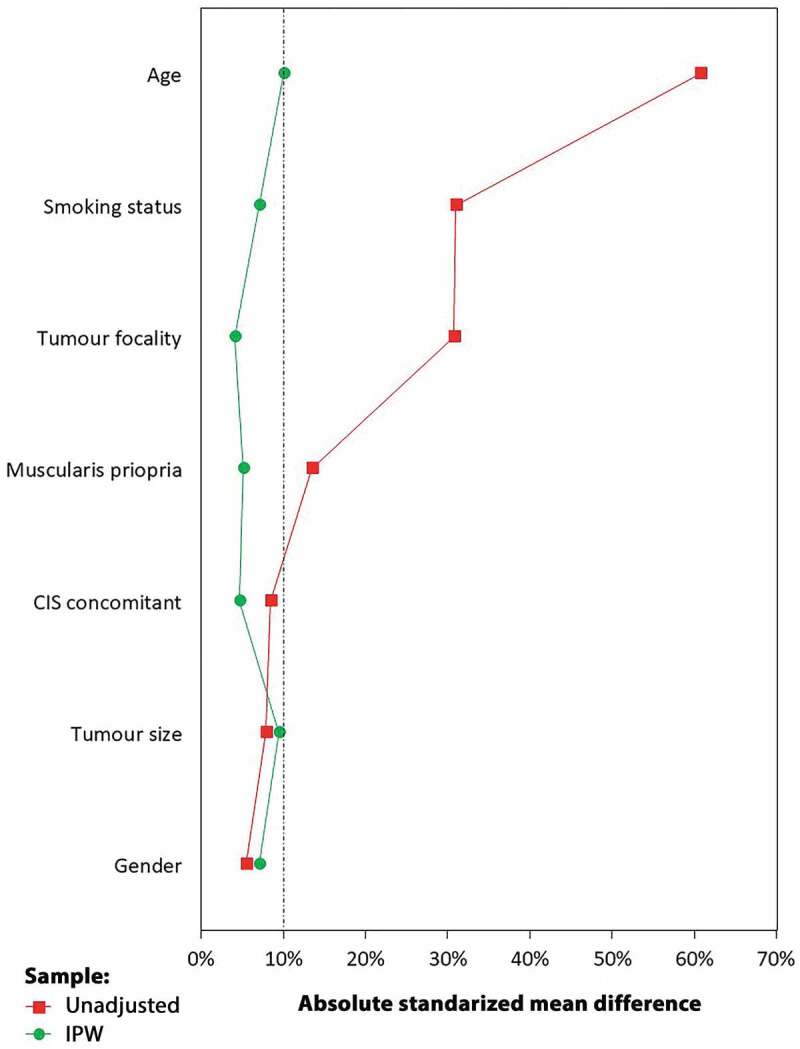
Covariates balance before adjustment and after IPW

To reduce the bias of unweighted estimators and adjust for covariates imbalance between groups without losing the patients, we performed complementary analysis using IPW. After IPW adjustment, absolute standardised mean differences for all adjusted clinicopathological variables were <10%, which indicated that patients in all strain groups were subsequently comparable (figure 2).

**Figure 3.**
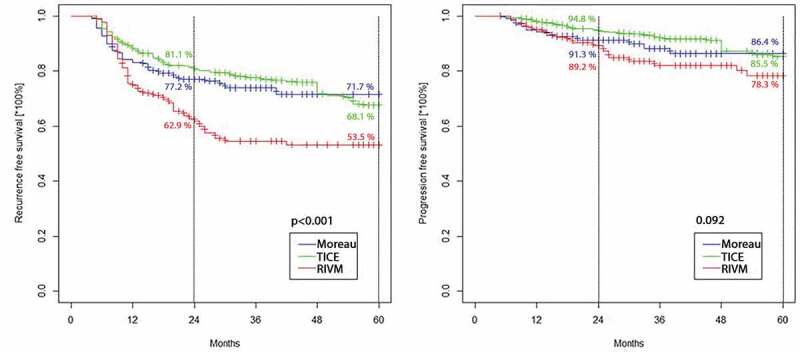
**A**. RFS for the 5-year follow-up after IPW (*P* < 0.001). **B**. PFS for the 5-year follow-up after IPW (*P* = 0.092)

The IPW-adjusted Cox proportional hazard regression analysis did not show any difference in RFS between the Moreau and TICE groups (HR 0.91, 95% CI 0.55–1.49 for TICE; *P* = 0.69), whereas the RIVM strain was significantly associated with worse RFS compared to the Moreau (HR 1.69, 95% CI 1.03–2.78 for RIVM; *P* = 0.034) and TICE strains (HR 1.87, 95% CI 1.25–2.81 for RIVM; *P* = 0.002) (figure 3A). In terms of PFS, the IPW-adjusted Cox proportional hazard regression analysis did not show any statistical difference between the Moreau and TICE groups (HR 0.74, 95% CI 0.37–1.47 for TICE; *P* = 0.399), as well as the Moreau and RIVM groups (HR 1.34, 95% CI 0.67–2.57 for RIVM; *P* = 0.413). Also, no statistical difference in PFS was observed between the TICE and RIVM groups (HR 1.79, 95% CI 0.94–3.38 for RIVM; *P* = 0.071) (figure 3B).

## Discussion

The original BCG strain was developed by Calmette and Guérin in 1921 from an attenuated strain of *Mycobacterium bovis*. During the following decades the sub-culturing process in various laboratories all over the world resulted in genetic evolution of the original strain [3]. Subsequently, results of several studies based on animal and *in vitro* models indicated that certain BCG sub-strains might differ in terms of oncological outcomes [7,8].

In the present study, a uniform group of patients with primary T1HG tumours was retrospectively analysed. We compared the oncological outcomes between three different BCG strains, including the Moreau, TICE and RIVM strains. Although study groups differed initially in terms of some clinicopathological variables (such as tumour size, tumour focality, presence of MP in the initial TURB specimen), which was primarily related to multicentric data collection and presence of some missing data, potential bias resulting from covariates imbalance was minimised after implementation of IPW. In the analysis of the study population (both IPW-unadjusted and -adjusted) significant differences in RFS were found between particular strain groups. Patients treated with the RIVM strain were more likely to experience disease recurrence compared to the Moreau or TICE strains; however, no significant differences in PFS were observed between any of the strains.

The BCG strains analysed in our present study have rarely been reported in the literature or they have not been directly compared yet in patients with T1HG NMIBC. D’Andrea *et al*. [9] in their retrospective study did not show any difference in RFS and PFS between the TICE and Moreau strains. Although primary analysis was conducted for the heterogeneous population of patients with different NMIBC stages, additional exploratory analysis adjusted for pathological stage (including the T1 subgroup) also revealed no association with RFS and PFS between the TICE and Moreau strains. Interestingly, a trend in RFS in favour of Moreau was observed if adequate BCG treatment was administered, but patients receiving BCG-Moreau were more likely to experience disease recurrence without adequate maintenance treatment [9]. On the contrary, we observed a slight trend towards BCG-TICE (HR 0.91 for TICE).

The Dutch Cooperative Trial evaluated mitomycin vs BCG-TICE vs BCG-RIVM in 469 patients with pTA/pT1 NMIBC and CIS of the urinary bladder after TURB, and no significant difference in terms of recurrence between analysed strains was found [10]. Contrary to that study, our present results indicated the possible inferiority of the RIVM strain.

Although several randomised controlled trials (RCTs) reported differences between various BCG strains, they were generally flawed by a small sample size and/or not involving patients receiving maintenance treatment [11–13]. However, because of the fact that maintenance BCG provides superior RFS benefit when compared to an induction course only, results from these trials may not be applicable to current practices. One example is a study by Rentsch *et al*. [14]. In their RCT including 132 patients with high-risk NMIBC, who received either BCG-Connaught (*n* = 71) or BCG-TICE (*n* = 60), the authors found a significantly higher 5-year RFS rate for patients treated with BCG-Connaught. However, no statistically significant difference was observed for PFS and overall survival. Additionally, the authors evaluated immunogenicity of the two strains in a mouse model and found that BCG-Connaught induced more robust T-cell recruitment to the bladder compared with BCG-TICE (*P* < 0.05), which might explain the differential efficacy of the Connaught and TICE strains. Even though the study was well-designed, it has to be once more highlighted that patients received only a BCG induction course [14].

Finally, a meta-analysis of prospective RCTs and recent network meta-analysis including 10 different BCG strains did not confirm the superiority of any BCG strain over any other [15,16]. Similarly, recent data from a prospective study conducted by Unda-Urzaiz *et al*. [17], as well as data from a *post hoc* analysis of a large Phase II prospective trial assessing BCG and interferon-α in both BCG-naive and BCG-failure patients [18] did not present any clear differences in oncological outcomes between various BCG strains. However, the quality of data (Level of Evidence 2a) did not allow for the drawing of definitive conclusions.

To our best knowledge, we directly compared oncological outcomes of different BCG strains in the largest cohort of patients with T1HG tumours to date. Strains presented in the present study, especially Moreau, are seldom reported in other trials. Also, patients included in our present study received a maintenance course, which was rarely presented in previous reports. Finally, contrary to the majority of other papers, both RFS and PFS were analysed in the present study. Despite several strengths, the present study has certain limitations. First, some centres collected clinical and pathological data prospectively, but most data were collected retrospectively. Thus, to overcome limitations of a retrospective design, as well as existing differences in baseline patients’ characteristics, we performed IPW-adjusted analyses. Second, the data included in the present study was mainly gathered from outpatient BCG departments. Therefore, meticulous details on patients who were not qualified or were dropped from BCG were not recorded and therefore not included in the analysis. Third, to preserve the homogeneity of the population, we included only patients that received at least five induction and two maintenance instillations, representing an adequate BCG exposure. However, this might mean that some relevant patients (e.g. patients with poor outcome at re-TURB after BCG induction) were not included. Fourth, there was no central specimens review and no substaging of T1 tumours. Fifth, all data used in the present paper originated from high-volume oncological centres, thus the results of this study may not be applicable to centres with lesser experience in bladder cancer treatment. Sixth, data regarding the experience of the surgeons, technique details were not available and therefore were not included in the analysis. Also, data about the WHO 1973 grade, immediate single-instillation chemotherapy, LVI, VH and prostatic involvement of the tumours were not uniformly reported and/or were unreliable, and therefore not included in the analysis. Finally, we did not perform cancer-specific survival analysis because the number of events (cancer-specific deaths) was low, and therefore, not statistically representative.

## Conclusions

The results of the present study suggest that the Moreau and TICE BCG strains might be superior to the RIVM strain in terms of RFS in patients with T1HG NMIBC.
